# Health care are associated with worsening of frailty in community older adults

**DOI:** 10.11606/S1518-8787.2019053000829

**Published:** 2019-03-26

**Authors:** Jair Almeida Carneiro, Cássio de Almeida Lima, Fernanda Marques da Costa, Antônio Prates Caldeira

**Affiliations:** I Universidade Estadual de Montes Claros. Centro de Ciências Biológicas e da Saúde. Departamento de Saúde Mental e Saúde Coletiva. Montes Claros, MG, Brasil; II Centro Universitário FIPMoc de Montes Claros. Montes Claros, MG, Brasil; III Universidade Estadual de Montes Claros. Centro de Ciências Biológicas e da Saúde. Programa de Pós-Graduação em Ciências da Saúde. Montes Claros, MG, Brasil; IV Universidade Estadual de Montes Claros. Centro de Ciências Biológicas e da Saúde. Departamento de Saúde da Mulher e da Criança. Montes Claros, MG, Brasil

**Keywords:** Aged, Frail Elderly, Risk Factors, Quality of Health Care, Surveys and Questionnaires, utilization, Idoso, Idoso Fragilizado, Fatores de Risco, Qualidade da Assistência à Saúde, Inquéritos e Questionários, utilização

## Abstract

**OBJECTIVE:**

To identify the factors associated with the worsening of frailty in older adults resident in the community.

**METHODS:**

This is a prospective, longitudinal, and analytical study. The data collection in the baseline occurred in the participants’ homes from a random sampling by conglomerates. Demographic and socioeconomic variables, morbidities, and use of health services were analyzed. Frailty was measured by the Edmonton Frail Scale. The second data collection was performed after an average period of 42 months. The adjusted prevalence ratios were obtained by multiple Poisson regression analysis with robust variance.

**RESULTS:**

A total of 394 older adults participated in both phases of the study, with 21.8% of them presenting worsening of the frailty condition. The variables that remained statistically associated with the transition to a worse state of frailty were: polypharmacy, negative self-perception of health, weight loss, and hospitalization over the past 12 months.

**CONCLUSIONS:**

The factors associated with worsening of frailty along the studied period among older adults in the community were those related to health care. This result must be considered by health professionals when addressing frail and vulnerable older adults.

## RESUMO

**OBJETIVO:**

Identificar os fatores associados à piora da fragilidade em idosos residentes na comunidade.

**MÉTODOS:**

Trata-se de um estudo longitudinal prospectivo e analítico. A coleta de dados na linha de base ocorreu no domicílio dos participantes a partir de uma amostragem aleatória por conglomerados. Foram analisadas variáveis demográficas e socioeconômicas, morbidades e utilização de serviços de saúde. A fragilidade foi aferida pela Escala de Fragilidade de Edmonton. A segunda coleta de dados foi realizada após um período médio de 42 meses. As razões de prevalências ajustadas foram obtidas por análise múltipla de regressão de Poisson com variância robusta.

**RESULTADOS:**

Participaram de ambas as etapas do estudo 394 idosos, sendo que 21,8% deles apresentaram piora da condição de fragilidade. As variáveis que se mantiveram estatisticamente associadas à transição para um estado pior de fragilidade foram: polifarmácia, autopercepção negativa de saúde, perda de peso e internação nos últimos 12 meses.

**CONCLUSÕES:**

Os fatores que se mostraram associados à piora da fragilidade ao longo do período estudado entre idosos na comunidade foram os relacionados aos cuidados de saúde. Esse resultado deve ser considerado pelos profissionais de saúde na abordagem ao idoso frágil e vulnerável.

## INTRODUCTION

Population aging is currently considered one of the greatest challenges for public health. This phenomenon is happening gradually in developed countries, but presents an accelerated rate in developing countries, leading to new challenges for the health sector ^1^ .

Aging involves gradual and inevitable changes. It is characterized as a dynamic, progressive, and irreversible process, linked to biological, psychological, and social factors ^2^ . The cumulative effect of several health-related conditions can increase susceptibility to disease and compromise the functional capacity of older adults to perform everyday activities and, consequently, lead to adverse clinical outcomes. The clinically recognizable state resulting from the decline in the reserve and function of multiple physiological systems is defined as frailty ^3^ .

Frailty can be observed in older adults who present physiological vulnerability to maintain or regain homeostasis after occurrence of stressor events. It is the reduction of energy reserves arising from changes related to the aging process, composed by sarcopenia, neuroendocrine dysregulation, and immunologic dysfunction. Homeostasis decompensation arises when acute, physical, psychological, or social events are able to lead to the increase of deleterious effects on the different organic systems of frail older adults ^3^ ^,^ ^4^ .

Simple, valid, accurate, and reliable instruments to identify frailty are necessary and important for administrators and health professionals. Researchers at the University of Alberta, in Edmonton, Canada, developed the Edmonton Frail Scale (EFS), which covers nine domains: cognition, general health status, functional independence, social support, medication use, nutrition, humor, urinary continence, and functional performance. It is considered a comprehensive scale, easy to handle and apply ^5^ . Culturally adapted to the Portuguese language in Brazil, the EFS is considered reliable and can be applied even by professionals who are not specialized in geriatrics or gerontology. The contents, construct, and criterion validity of the adapted instrument has already been confirmed ^6^ .

Serial frailty evaluations in older adults show the dynamic process of this condition^7–11^, revealing the transition between the states of frailty over time. However, little is known about the relevance of this process in Brazil, where these individuals are exposed to different social, economic, functional, and social assistance contexts, capable of influencing the transition between different levels of frailty. Changes in health states are considered to be of clinical and public health interest. Recognizing the factors that can aggravate this state is important for the development of measures that may prevent its progression. This study aimed to identify the factors associated with the worsening of frailty in older adults residents in the community.

## METHODS

This is a prospective, longitudinal and analytical study, with a populational and household base, with a quantitative approach, carried out with community older adults. The research was developed in a medium-sized municipality in the state of Minas Gerais, southeastern Brazil. The city has a population of approximately 400 thousand inhabitants and is the main urban pole of the region.

The baseline sample size was calculated to estimate the prevalence of each health outcome investigated in the epidemiological investigation. An estimated population of 30,790 older adults residents in the urban region, according to data from the Brazilian Institute of Geography and Statistics (IBGE), a 95% confidence level, a conservative prevalence of 50% for the unknown outcomes, and a sampling error of 5% were considered. As the sampling was made by conglomerates, the identified number was multiplied by a correction factor and design effect ( *deff* ) of 1.5% plus 15% for any possible losses. The minimum number of older adults defined by sample calculation was 656 individuals ^12^ .

Between May and July 2013 (base year), 685 older adults aged 60 years or more were allocated to the study. Aiming to continue the research, the first wave of the study was carried out between the months of November 2016 and February 2017. In this step, the residences of all older adults interviewed in the base year were considered eligible for the new interview. We considered as losses the individuals who were not available to participate of at least three visits on different days and times, even with prior scheduling, as well as those who changed the place of residence and those who died. The individuals who changed place of residence were not sought because there was no information on the current address. We used the same data collection instruments previously employed, all validated ^12^ .

The dependent variable was the transition record for a worse state of the EFS component. The scale has nine domains, distributed in 11 items with scores from zero to 17. The score can vary between: zero and four, indicating that there is no presence of frailty; five and six, defining the older adult as apparently vulnerable; seven and eight, indicating mild frailty; nine and 10, moderate frailty; and 11 or more, severe frailty ^5^ . Classification results of each older adult were compared between the two phases of the study (first wave and baseline).

For data analysis, the results of the dependent variable were dichotomized at two levels: worsening and non-worsening of the general EFS score. The independent variables studied were also dichotomized: sex (male or female); age group (up to 79 years or ≥ 80 years); marital status (with companion, including married and stable union, or without companion, including single, widowed, and divorced); family arrangement (lives alone or does not live alone); education level (up to four years of study or more than four years of study); literacy (knows how to read or not); religious practice (yes or no); has his/her own income (yes or no); monthly household income (up to a minimum wage or more than one minimum wage); presence or absence of chronic diseases (hypertension, diabetes mellitus, heart disease, osteoarthritis, neoplasm, stroke); depressive symptoms, according to the reduced version of the Geriatric Depression Scale by Yesavage (GDS-15), in which a score equal to or greater than six identifies depressive symptoms ^13^ ; polypharmacy, defined as using five or more medication (yes or no); self-referred weight loss (yes or no); smoking (yes or no); presence of caregiver (yes or no); fall in the past 12 months (yes or no); medical consultation in the last 12 years (yes or no); hospitalization in the past 12 months (yes or no); and self-perception of health. This last variable was evaluated through the question: “How would you classify your health status?”, with possible answers being “very good”, “good”, “regular”, “bad”, or “very bad”. For analysis, the answers “very good” and “good” were assumed as positive health perceptions, while the answers “regular”, “bad”, and “very bad” were classified as negative health perceptions, following similar studies on the theme ^14^ ^,^ ^15^ .

Bivariate analyses were performed to identify factors associated with the variable answer by chi-square test. The magnitude of the associations was estimated from the prevalence reasons (PR). Poisson regression, with robust variance, was used to calculate the adjusted PR, considering, jointly, the independent variables that were more strongly associated with the worsening of the EFS component in the bivariate analysis, up to a 20% significance level (p < 0.20). For the final analysis, a significance level of 0.05 was considered (p < 0.05).

The chi-square test was also used to compare the main characteristics between the accompanied group and losses. The information collected were analyzed by the Statistical Package for the Social Sciences (SPSS) software, version 17.0 (SPSS for Windows, Chicago, USA).

All participants were instructed concerning the research and showed their consent by signing an informed consent form. The research project was approved by the Research Ethics Committee through the Embodied Opinion no. 1.629.395.

## RESULTS

Among the 685 older adults evaluated in the base year, 92 refused to participate in the second phase of the study, 78 changed their residence and have not been located, 67 were not found at home after three visits, and 54 had died. Thus, 394 older adults participated in this study.

Table 1 presents a comparison of the characteristics in the base year between the accompanied population of older adults and the population of older adults considered as loss during this study’s follow-up. We did not find any significant differences for the main characteristics of the groups, which highlights a non-differential loss.

**Table 1:** Comparison of the main characteristics between accompanied and lost older adults in the study’s first wave of follow-up in a municipality in Minas Gerais, Brazil, 2013–2017.

Variable	Accompanied		Follow-up losses		p
	
	n	%	n	%	
Sex					0.163
Male	130	33.0	111	38.1	
Female	264	67.0	180	61.9	
Age group (years)					0.089
< 80	341	86.5	238	81.8	
≥ 80	53	13.5	53	18.2	
Education level					0.964
≤ 4 years	300	76.1	222	76.3	
> 4 years	94	23.9	69	23.7	
Monthly household income					0.158
≤ 1 minimum wage	121	30.7	75	25.8	
> 1 minimum wage	273	69.3	216	74.2	
Arterial hypertension					0.937
Yes	280	71.1	206	70.8	
No	114	28.9	85	29.2	
Diabetes mellitus					0.137
Yes	80	20.3	73	25.1	
No	314	79.7	218	74.9	
Depressive symptoms					0.870
Yes	116	29.4	84	28.9	
No	278	70.6	207	71.1	
Polypharmacy					0.229
Yes	86	21.8	75	25.8	
No	308	78.2	216	74.2	
Frailty					0.209
Frail	132	33.5	111	38.1	
Not frail	262	66.5	180	61.9	

Regarding the transition between the EFS components, 21.8% of the older adults presented worsening of the frailty state, 33.0% showed improvement, and 45.2% showed no change ( Table 2 ). The prevalence of frailty went from 33.8% in the base year to 36.8% in this first wave of the study.

**Table 2:** Transition between levels of frailty, according to the Edmonton Frail Scale (EFS), from the baseline to the first wave of the study in a municipality of Minas Gerais, Brazil, 2013–2017.

Baseline			First wave									
	
Levels of frailty (EFS)			No frailty		Apparently vulnerable		Mild frailty		Moderate frailty		Severe frailty	
					
	n	%	n	%*	n	%*	n	%*	n	%*	n	%*
No frailty	174	44.2	125	71.8	32	18.4	14	8.0	3	1.7	0	0.0
Apparently vulnerable	87	22.1	40	46.0	25	28.7	17	19.5	5	5.7	0	0.0
Mild frailty	73	18.5	21	28.8	20	27.4	21	28.8	11	15.1	0	0.0
Moderate frailty	39	9.9	3	7.7	11	28.2	15	38.5	6	15.4	4	10.3
Severe frailty	21	5.3	1	4.8	5	23.8	7	33.3	7	33.3	1	4.8

* The percentages refer to the proportion of each level of frailty regarding the baseline.

Tables 3 and 4 show the results of the bivariate analysis between the characteristics of the older adults and the worsening of level of frailty. No sociodemographic variable proved to be associated with worsening of frailty.

**Table 3:** Associations between the transition to worse levels of the Edmonton Frail Scale components and demographic, social, and economic variables of older adults residents in the community accompanied in the first wave of the study (bivariate analysis) in a municipality of Minas Gerais, Brazil, 2013–2017.

Independent variable	Transition to worse levels of frailty						

	Yes		No		PR	95%CI	p

	n	%	n	%			
Sex							0.097
Male	35	26.7	96	73.3	1.06	0.98–1.14	
Female	51	19.4	212	80.6	1		
Age group (years)							0.981
< 80	66	21.9	236	78.1	1.00	0.92–1.08	
≥ 80	20	21.7	72	78.3	1		
Marital status							0.402
With partner	46	23.6	149	76.4	1.02	0.96–1.10	
Without partner	40	20.1	159	79.9	1		
Family arrangement							0.691
Lives alone	12	24.0	38	76.0	1.02	0.92–1.12	
Does not live alone	74	21.6	270	78.4	1		
Education level							0.463
≤ 4 years	67	22.7	228	77.3	1.03	0.95–1.11	
> 4 years	19	19.2	80	80.8	1		
Knows how to read							0.664
Yes	67	22.3	233	77.7	1.01	0.94–1.10	
No	19	20.2	75	79.8	1		
Religious practice							0.140
No	5	38.5	8	61.5	1.14	0.94–1.38	
Yes	81	21.3	300	78.7	1		
Has his/her own income							0.842
No	9	23.1	30	76.9	1.01	0.90–1.13	
Yes	77	21.7	278	78.3	1		
Monthly household income							0.446
≤ 1 minimum wage	25	24.5	77	75.5	1.02	0.95–1.11	
> 1 minimum wage	61	20.9	231	79.1	1		

**Table 4:** Associations between the transition to worse levels of the Edmonton Frail Scale components and variables related to morbidities and use of health services among older adults residents in the community accompanied in the first wave of the study (bivariate analysis) in a municipality of Minas Gerais, Brazil, 2013–2017.

Independent variable	Transition to worse levels of frailty						

	Yes		No		PR	95%CI	p
	
	n	%	n	%			
Arterial hypertension							0.323
Yes	65	23.1	216	76.9	1.03	0.96–1.11	
No	21	18.6	92	81.4	1		
Diabetes mellitus							0.494
Yes	22	24.4	68	75.6	1.02	0.94–1.11	
No	64	21.1	240	78.9	1		
Heart disease							0.998
Yes	24	21.8	86	78.2	1.00	0.92–1.07	
No	62	21.8	222	78.2	1		
Osteoarticular disease							0.670
Yes	43	22.8	146	77.2	1.01	0.95–1.08	
No	43	21.0	162	79.0	1		
Osteoporosis							0.552
Yes	34	23.4	111	76.6	1.02	0.95–1.09	
No	52	20.9	197	79.1	1		
Neoplasm							0.018
Yes	14	36.8	24	63.2	1.13	1.01–1.27	
No	72	20.2	284	79.8	1		
Stroke							0.087
Yes	10	34.5	19	65.5	1.11	0.97–1.27	
No	76	20.8	289	79.2	1		
Polypharmacy							0.001
Yes	36	33.6	71	66.4	1.13	1.05–1.22	
No	50	17.4	237	82.6	1		
Self-perception of health							< 0.001
Negative	62	30.0	145	70.0	1.15	1.08–1.22	
Positive	24	12.8	163	87.2	1		
Weight loss							< 0.001
Yes	25	42.4	34	57.6	1.20	1.09–1.32	
No	61	18.2	274	81.8	1		
Has a caregiver							0.716
Yes	11	23.9	35	76.1	1.01	0.91–1.13	
No	75	21.6	273	78.4	1		
Fall in the last 12 months							0.016
Yes	36	29.3	87	70.7	1.09	1.01–1.17	
No	50	18.5	221	81.5	1		
Medical consultation in the last 12 months							0.293
Yes	81	22.5	279	77.5	1.06	0.95–1.19	
No	05	14.7	29	85.3	1		
Hospitalization in the last 12 months							< 0.001
Yes	25	43.9	32	56.1	1.21	1.10–1.34	
No	61	18.1	276	81.9	1		

The variables that remained statistically associated with the transition to a worse state of frailty after multiple analysis, according to the EFS, were: polypharmacy, negative self-perception of health, weight loss, and hospitalization over the past 12 months ( Table 5 ).

**Table 5:** Factors associated with the worsening of frailty in the older adults residents in the community (multiple analysis) in a municipality of Minas Gerais, Brazil, 2013–2017.

Independent variable	PR	95%CI	p
Polypharmacy			0.030
Yes	1.08	1.01–1.17	
No	1		
Self-perception of health			< 0.001
Negative	1.13	1.06–1.20	
Positive	1		
Weight loss			0.003
Yes	1.15	1.05–1.27	
No	1		
Hospitalization in the last 12 months			0.003
Yes	1.16	1.05–1.27	
No	1		

## DISCUSSION

This study showed the transition through different levels of frailty in community older adults in a period of 42 months between the baseline and the first wave of the study, identifying that the conditions associated with the worsening of the state of frailty were related to morbidities and negative self-perception of health. The prevalence of frailty increased slightly between the two moments. Almost half of the older adults group maintained the standard of the previous evaluation and about a fifth presented worsening of frailty, while others showed improvement, which highlights the dynamic process of frailty, as already pointed out by others authors ^7^ ^,^ ^8^ .

A pioneer study ^7^ noted, in three equal intervals during 54 months, that the transitions to states of greater frailty were more frequent, with 43.3% rates, than transitions to states of less frailty, with rates of up to 23.0%, while the transition from frail to not frail was low, with rates from 0% to 0.9%. Another study ^8^ showed the following transition of frailty in 24 months: 22.1% of individuals were worse, 61.8% showed no change, and 16.1% have improved.

The variations in the percentages found in the studies can be justified by the use of different instruments with different parameters or by the lack of methodological standardization, with differences in the intervals of time between evaluations and in the composition of the sample, including age group. Although a number of instruments to assess frailty in older adults exist, the reliability and validity of the majority have not yet been evaluated.

A systematic review with 150 studies registered 27 different instruments to evaluate frailty in older adults. Among them, only two, including the EFS, followed the guideline that discusses the best practices in the development of complex measures. Such instruments presented acceptable reliability and good concurrent predictive validity ^16^ .

The results of this study show a significant association between worsening of frailty in older adults living in the community with negative self-perception of health. This relationship was also identified in a systematic review of longitudinal studies on the subject ^17^ . The self-perception of health, a subjective and comprehensive measure of health condition, is an indicator able to evaluate health in an effective, quick, and low-cost way, as well as the life context of populational groups ^18^ . It incorporates physical, cognitive, and emotional components as aspects related to the well-being and satisfaction with their own life ^19^ ^,^ ^20^ . This measure has, therefore, the ability to predict robustly and consistently the worsening of frailty in older adults, in addition to mortality and decline of functional ability ^20^ ^,^ ^21^ .

The results showed an association between the worsening of frailty and weight loss, a frequently used marker to evaluate nutrition. The compromised nutritional status is an important determinant related to frailty in older adults, as evidenced in two systematic reviews on the theme, which highlighted the association between malnutrition (measured by weight loss) and frailty ^17^ ^,^ ^22^ . The adequate intake of macro and micronutrients is able to delay the beginning of negative effects promoted by frailty in older adults, as well as slow down its development and progression. The quality of the diet is inversely associated with the risk of being frail ^22^ .

The worsening of frailty in older adults residents of the community was associated to polypharmacy, as also identified in another study ^10^ . The vulnerability of older adults to the problems arising from the use of medicines is significant, due to the complexity of the clinical conditions, the need of various therapeutic agents, and pharmacokinetic and pharmacodynamic characteristics inherent to aging, in addition to the potential adverse effects of medication interaction ^23^ . The use of many medication can expose older adults to vulnerability to stressors events, represented by the organism’s difficulty in restoring homeostasis, thus predisposing to worsening of frailty.

This worsening was also associated with hospitalization in the last 12 months, as noted in other research ^9^ ^,^ ^11^ . This fact reinforces the importance of rehabilitation after hospitalization as a measure to reduce the deterioration of older adults’ health conditions. This aspect is particularly important for health professionals that care for this population. Hospitalizations, even when short, tend to interfere a lot in the older adults’ daily life, precipitating changes that make them more frail. Therefore, family health strategy teams should establish closer ties with these individuals, providing fast recovery and anticipating worsening situations, such as inadequacies or interruptions in the use of medication and physical inactivity, among others.

Some factors were associated with worsening of frailty in other investigations^8–11^ and highlighted in systematic review of longitudinal studies ^17^ , but were not present in this study. In Europe ^8^ , data from 14,424 people residing in the community with age equal to or greater than 55 years were analyzed. Older adults aged over 65 years, of the female sex, and with low educational level presented higher risk of worsening of frailty. In 3,018 Chinese individuals aged 65 years or more, who lived on the community ^9^ , hospitalization, advanced age, previous stroke, previous cancer, diabetes mellitus, and osteoarthritis were associated with worsening of frailty. Social and behavioral factors, including smoking, were associated to the worsening of frailty in study in North-American individuals ^24^ . In Italian older adults ^10^ , socioeconomic and clinical factors were related to worsening of frailty, including older age, female sex, obesity, cardiovascular disease, osteoarthritis, smoking, and polypharmacy. The observed differences probably depict the particularities presented by each populational group, in addition to the different time intervals and instruments to evaluate frailty used by the authors.

Some studies investigated and observed that pulmonary diseases ^9^ , consumption of alcoholic drinks ^24^ , loss of vision, hypovitaminosis D and hyperuicemia ^10^ , as well as low cognitive function ^9^ ^,^ ^10^ are also factors associated with worsening of frailty. However, these factors were not investigated in this study, which represents a limitation to a comparative analysis.

Another limitation was the inability to evaluate frailty transitions that have occurred at shorter time intervals than the period between the baseline and the first wave of the study. In addition, part of the variables studied were self-reported. However, despite these limitations, this study has a random sample, with large number of older adults living in the community. We used an instrument previously adapted to the Brazilian culture, standardized, comprehensive, and with simple, valid, accurate, and reliable methods. In addition, this study addressed a range of variables that potentially influence the transitions of frailty, highlighting aspects that can be used by administrators and health professionals.

Finally, we concluded that frailty was confirmed as a dynamic process, characterized by transition between levels of the scale over time. Health-related factors that showed association with worsening of frailty in older adults living in the community were: polypharmacy, negative self-perception of health, weight loss, and hospitalization over the past 12 months. These results should be considered by health professionals who deal with older adults, especially family health strategy teams. Recognizing them as markers of worsening of the state of frailty, health professionals can make timely interventions to prevent the aggravation of older adults’ health conditions and promote better quality of life.
